# Bone health in asthma: a twofold effect of disease and pharmacotherapy

**DOI:** 10.3389/fendo.2026.1799396

**Published:** 2026-06-02

**Authors:** Sidian Yang, Yihao Wang, Xingchao Li, Yewen Niu, Liming Lei, Zichao Han

**Affiliations:** 1 Shandong University of Traditional Chinese Medicine, Jinan, Shandong, China; 2 The First People’s Hospital of Jinan, Jinan, Shandong, China; 3 The People’s Hospital of ZouPing City, Binzhou, Shandong, China

**Keywords:** asthma, bone health, glucocorticoids, inflammation, vitamin D

## Abstract

Asthma is a chronic inflammatory airway disease with high global prevalence. Its persistent systemic inflammation and long-term treatments pose significant risks to bone health. This article aims to systematically elucidate the mechanisms by which asthma affects bone health and the corresponding clinical management strategies. Chronic inflammation disrupts bone metabolism through immune dysregulation, pro-inflammatory cytokine release, and oxidative stress, enhancing bone resorption and suppressing formation. Disease-related physical inactivity and common vitamin D (VitD) deficiency further impair bone health by reducing mechanical loading and altering endocrine regulation. Pharmacologically, long-term or high-dose glucocorticoids (GCs) therapy significantly lowers bone mineral density and increases fracture risk. Based on these mechanisms, comprehensive management strategies include optimizing inhaled therapies and judicious use of natural medicines to limit GCs exposure. Future research should clarify molecular mechanisms of the lung–bone axis, develop risk assessment models integrating inflammatory markers and cumulative GCs exposure, and implement routine bone health monitoring for high-risk populations. Multidisciplinary collaboration among respiratory medicine, paediatrics, and orthopaedics is essential to balance asthma control with preservation of bone health.

## 1 Introduction

Asthma is a highly prevalent chronic respiratory disease affecting approximately 260 million individuals worldwide, posing a major public health burden (1). Beyond intermittent bronchospasm, asthma involves persistent airway inflammation that drives airway hyperresponsiveness and structural remodelling, clinically manifesting as reversible airflow limitation and requiring long-term management (2). Inhaled corticosteroids (ICS) are first-line therapy per Global Initiative for Asthma (GINA) guidelines, effectively reducing exacerbations. In severe cases, long-term or repeated oral corticosteroid (OCS) use is often necessary (3). Prolonged systemic corticosteroid exposure raises safety concerns, particularly for skeletal health. In children, chronic inflammation exacerbates corticosteroid-related adverse effects on bone development, creating a dual insult that can reduce bone mineral density, impair microarchitecture, increase fracture risk, and compromise long-term quality of life (4, 5). Although previous studies have examined asthma-related inflammation and GCs therapy on bone separately, a systematic framework for their combined effects is lacking. This review proposes a two-hit model, in which systemic inflammation and therapeutic GCs exposure act as independent, additive skeletal risk factors. Using this framework, we synthesize underlying mechanisms, clinical evidence, and management strategies, highlight research gaps and future directions, and provide a theoretical foundation and practical guidance for interdisciplinary practice and research in respiratory medicine, paediatrics, and orthopaedics.

## 2 Bone development and repair

Bone development and repair depend on the dynamic balance between osteoblast-mediated bone formation and osteoclast-mediated bone resorption (6). During embryonic development, endochondral ossification predominates. Mesenchymal stem cells (MSCs) first differentiate into chondrocytes to form a cartilage anlage. Following vascular invasion, MSCs become osteoblasts, which secrete type I collagen-rich bone matrix and initiate mineralisation (7–9). The epiphyseal cartilage, or growth plate, drives longitudinal bone growth in childhood and adolescence. Its layered structure, including proliferative, hypertrophic, and calcification zones, supports ongoing cartilage proliferation, hypertrophy, and replacement by bone. Bone width increases through periosteal apposition, in which osteoblasts deposit outer lamellae, balanced by osteoclast resorption of inner trabeculae and subsequent osteoblastic repair (10). In adulthood, longitudinal growth ceases, but bone homeostasis continues through basic multicellular units. Osteoclast-mediated resorption is followed by osteoblast deposition and mineralisation, maintaining bone density and repairing microdamage (11). Bone repair is categorised by injury severity into physiological and pathological processes. Physiological bone repair depends on remodelling, in which osteocytes first sense microdamage or injury and signal for osteoclast recruitment to resorb the affected bone, after which osteoblasts deposit and mineralize new matrix to complete the repair process (12–14). The repair of pathological injuries such as fractures and bone defects occurs in four collaboratively regulated stages. In the inflammatory stage, a haematoma forms at the fracture site, releasing factors such as platelet-derived growth factor (PDGF) and transforming growth factor beta (TGF-β), which recruit neutrophils and macrophages to clear necrotic tissue and induce MSCs migration toward the injury. During the chondrogenesis stage, the hypoxic environment drives MSCs differentiation into chondrocytes, which secrete type II collagen to form a soft callus that temporarily stabilises the fracture site, while gradual vascular invasion begins. In the ossification stage, chondrocytes undergo hypertrophy and apoptosis, osteoblasts migrate in and deposit type I collagen, and the soft callus mineralises into a hard callus, substantially enhancing mechanical strength. In the remodelling stage, osteoclasts resorb disorganised woven bone, and osteoblasts reconstruct lamellar bone according to mechanical demands, ultimately restoring normal anatomical structure and mechanical function (15–18). Meanwhile, the efficiency of repair is governed by the functional status of MSCs, the activity of osteoblasts and osteoclasts, and key regulatory factors such as bone morphogenetic proteins (BMPs), vascular endothelial growth factor (VEGF), TGF-β, calcium, and VitD (9, 13, 19).

In summary, the shared mechanisms of skeletal development and repair can be captured in three core regulatory principles. First, the directed differentiation of MSCs provides the foundational process, guided by specific signalling pathways and supplying the primary precursors for bone and cartilage formation during both embryogenesis and post-injury repair. Second, vascular invasion and network formation establish the critical link between angiogenesis and osteogenesis, enabling embryonic ossification centre formation and delivering nutrients to sites of injury. Third, a precise balance between osteoblast and osteoclast activity determines functional outcomes, with their coordinated actions optimising bone structure during development and restoring mechanical strength following repair. Disruption of this balance can result in skeletal maldevelopment or impaired repair. Understanding these fundamental mechanisms offers a framework for exploring how asthma and its treatments may disturb this finely regulated system.

## 3 The dual impact of asthma and its treatment on skeletal health

**Figure 1:**
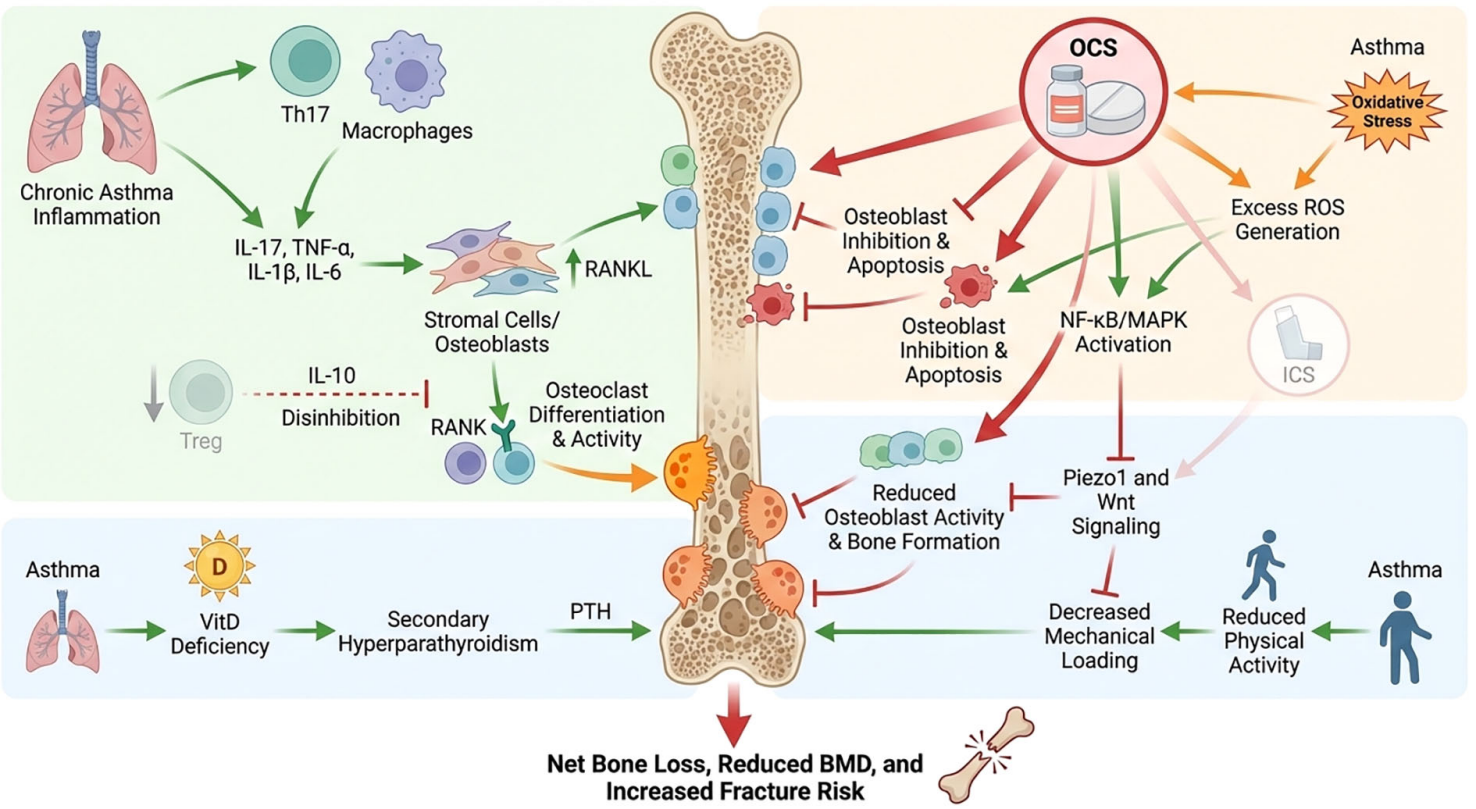
Schematic diagram of the pathological mechanism of asthma-related bone metabolic disorder. It systematically elucidates the pathological mechanisms by which asthma-associated chronic inflammation, oxidative stress, and GCs therapy synergistically disrupt the balance of bone metabolism through multiple signalling pathways, thereby leading to bone loss.

Asthma extends beyond a localized airway disorder and is increasingly recognised as a systemic inflammatory disease that directly and independently threatens skeletal health (20). Growing evidence indicates that reduced bone mineral density and elevated fracture risk remain significant concerns in patients with asthma, even without corticosteroid exposure. This association is especially pronounced in individuals with poorly controlled or severe disease, implying the presence of an intrinsic pathophysiological link driven by asthma itself (**Figure 1**) (21). The mechanisms underlying this link are discussed below.

### 3.1 Inflammation and oxidative stress

The chronic inflammatory state inherent to asthma serves as an independent risk factor for skeletal impairment, disrupting bone metabolic homeostasis through systemic inflammation, immune cell dysregulation, and downstream signalling pathways (22). A hallmark event in asthma pathogenesis is the binding of allergens to Immunoglobulin E (IgE), which cross-links high-affinity receptors on mast cells, triggering their activation and the subsequent release of diverse inflammatory mediators (23). Growing evidence suggests that many of these mast cell-derived mediators can negatively impact bone by promoting osteoclastogenesis and/or suppressing osteoblast activity (24). In the asthmatic microenvironment, overactivated T helper 17 cells (Th17) secrete Interleukin (IL)-17, while innate immune cells such as macrophages and dendritic cells release proinflammatory mediators including tumour necrosis factor-alpha (TNF-α), IL-1β, and IL-6. These cytokines act as potent osteoclast activating factors by upregulating RANK ligand (RANKL) expression, which binds to receptor activator of nuclear factor-κB (RANK) on osteoclast precursors and promotes their differentiation into mature osteoclasts, thereby enhancing bone resorption (25, 26). Simultaneously, the asthmatic environment impairs both the number and function of Regulatory T cells (Treg), reducing the secretion of anti-inflammatory mediators such as IL-10 and weakening their inhibitory effects on osteoclastogenesis. The resulting Th17/Treg imbalance collectively drives excessive bone resorption (27). During systemic inflammation, bone cells and monocytes release proinflammatory cytokines including IL-1β, TNF-α, IL-6, and IL-17. These mediators disrupt the Osteoprotegerin (OPG)/RANK/RANKL axis by increasing RANKL expression and altering OPG regulation, thereby enhancing osteoclast activity, accelerating bone resorption, and impairing bone strength (28, 29). Osteoporosis occurs more frequently in patients with COPD than in those with asthma, partly because COPD is associated with a more pronounced systemic inflammatory response (30).

Reactive oxygen species (ROS) are highly reactive oxygen-containing molecules with dual physiological roles. At normal levels, ROS act as intracellular second messengers, regulating gene expression, supporting cell survival, and modulating signalling networks, thereby influencing key cellular processes such as differentiation, migration, and proliferation (31, 32). Excessive ROS production, however, overwhelms antioxidant defences and induces oxidative stress, activating pro-osteoclastic pathways including nuclear factor kappa-B (NF-κB) and mitogen-activated protein kinase (MAPK), while directly impairing osteoblast function and promoting apoptosis (33, 34). Experimental studies show that ROS bidirectionally regulate osteoblast proliferation through the mechanistic target of rapamycin (mTOR) pathway. Low ROS levels activate mTORC1 and enhance proliferation, whereas high ROS levels inhibit mTOR via the AMP-activated protein kinase (AMPK)/Raptor pathway (35). In asthma, chronic airway inflammation drives excessive ROS generation, causing redox imbalance and further disrupting the balance between bone formation and resorption. ROS thus act as a molecular bridge linking airway inflammation to skeletal metabolic dysfunction (36). In summary, chronic inflammation in asthma establishes a pathological environment that promotes bone loss through systemic inflammatory mediators, immune dysregulation, and oxidative stress. These mechanisms help explain the decreased bone mineral density and increased fracture risk observed even in corticosteroid-naïve patients. Targeting inflammatory and oxidative pathways may therefore offer promising strategies to preserve skeletal health in this population.

### 3.2 VitD deficiency

VitD plays a central and multifaceted role in the interplay between asthma and skeletal health. Traditionally recognised as a key regulator of calcium homeostasis, VitD maintains bone integrity by promoting intestinal calcium absorption and supporting bone mineralisation (37). Epidemiological studies report a high prevalence of VitD insufficiency among asthma patients, with serum levels inversely correlated with allergy severity and positively correlated with lung function indices. A bidirectional relationship exists in which severe asthma is associated with lower VitD levels, while VitD deficiency increases the risk of severe asthma, underscoring the importance of monitoring VitD status in clinical assessment. Experimental evidence shows that VitD enhances the anti-inflammatory effects of GCs by upregulating MAPK Phosphatase 1 (MKP-1) expression and potentiating GCs receptor binding to DNA (38–40). In a gender-specific analysis, Lei et al. demonstrated protective effects of VitD intake against asthma, with an overall inverse association with asthma risk (OR = 0.92) and a more pronounced effect in males (OR = 0.79). The study also revealed a non-linear association between VitD intake and asthma mortality, suggesting optimal survival benefits within specific intake levels, providing insight for gender-tailored nutritional strategies (41). By contrast, meta-analyses indicate that in asthma patients already receiving standard pharmacotherapy, VitD supplementation does not confer additional benefits regarding exacerbation frequency, lung function, airway inflammation, or symptom control (42). It should be noted that the causal relationship between VitD levels and asthma severity remains incompletely understood. Whether reduced VitD levels directly contribute to asthma exacerbation or instead represent a concomitant phenomenon associated with severe disease, reduced outdoor activity, or long-term GCs therapy requires further clarification through prospective studies and randomized controlled trials. VitD deficiency can also trigger secondary hyperparathyroidism due to insufficient intestinal calcium absorption, leading to hypocalcaemia. Compensatory PTH secretion drives osteoclast differentiation and activation, resulting in bone loss and eventual osteoporosis (43, 44). These findings highlight VitD’s critical role in managing both asthma and skeletal health and suggest that future research should further elucidate its molecular mechanisms and explore personalised strategies tailored to VitD status.

### 3.3 Physical activity limitations

Regular exercise provides well-established clinical benefits for patients with asthma, including improved lung function, reduced airway hyperresponsiveness, fewer acute exacerbations, and enhanced quality of life (45). Extensive evidence supports both the safety and efficacy of regular exercise in this population (46). For instance, short-term exercise reduces IL-5, IL-6, and leptin levels in patients with well-controlled mild asthma, whereas it increases IL-13 levels in obese patients with asthma, suggesting phenotype-specific inflammatory responses (47). A meta-analysis further demonstrated that combined exercise modalities provide the greatest improvement in quality of life, with high-intensity interval training showing superior benefits compared with moderate-intensity continuous aerobic exercise, while exercise duration significantly influences therapeutic efficacy (48). Another meta-analysis in paediatric asthma found that exercise training improves exercise capacity and quality of life, although it does not significantly reduce FeNO levels, and improvements in lung function are largely limited to transient increases in FVC% and FEF 25-75% within eight weeks (49). Despite these benefits, reduced exercise tolerance and the risk of exercise-induced bronchospasm often cause patients with asthma to decrease physical activity voluntarily or involuntarily, thereby limiting access to these protective effects.

Asthma impairs bone formation and repair through exercise limitation, primarily by reducing mechanical loading and inducing secondary metabolic disturbances that disrupt bone metabolism. Patients with asthma often exhibit reduced exercise tolerance due to airway hyperresponsiveness, ventilatory limitation, and the risk of exercise-induced bronchoconstriction. Consequently, moderate-to-vigorous physical activity is frequently reduced, leading to chronically diminished mechanical stimulation of bone tissue (50). Mechanical loading is a key regulator of bone formation. It promotes osteoblast proliferation through activation of Piezo1 mechanosensitive ion channels, enhances MSCs osteogenic differentiation, and stimulates anabolic pathways such as BMPs and Wingless/Integrated (Wnt) signalling. Reduced physical activity in asthma directly impairs these regulatory mechanisms, resulting in decreased osteoblast activity, reduced bone matrix synthesis and mineralization, and slower bone formation (21, 51–53). Physical inactivity also disrupts the balance between osteoblasts and osteoclasts. Loss of mechanical stimulation alters the OPG/RANKL axis, enhances osteoclast activity, and accelerates bone resorption, leading to reduced bone density and deterioration of trabecular microarchitecture that weakens skeletal strength (54). During bone repair, asthma-associated activity restriction diminishes exercise-induced VEGF production, impairing vascular invasion and vascular network formation at injury sites (55). Inadequate vascularization limits blood supply, compromises callus mineralization and hard callus formation, and restricts delivery of osteogenic signals such as BMPs and TGF-β, thereby delaying the transition from cartilaginous to bony callus and weakening regenerated bone tissue (56, 57). Chronic physical inactivity further reduces secretion of anabolic hormones including growth hormone (GH) and insulin-like growth factor 1 (IGF-1) (58). GH promotes MSCs osteogenic differentiation and osteoblast function, whereas IGF-1 enhances bone matrix synthesis and callus maturation. Reduced levels of these hormones therefore further impair bone formation and repair, aggravating skeletal metabolic dysfunction (59, 60). Collectively, these mechanisms establish a pathological cascade through which asthma-related activity restriction progressively compromises skeletal development and bone repair capacity. It should be noted that activity limitation in children with asthma differs from that observed in adults. Children generally engage in higher-intensity daily physical activity and experience exercise-induced bronchospasm more frequently, making activity restriction potentially more pronounced in this population (61). In addition, treatment adherence in children is often suboptimal, and caregivers may excessively restrict physical activity because of concerns about symptom exacerbation, causing actual activity levels to fall below recommended standards (62). Nevertheless, children typically recover exercise capacity more rapidly following standardized treatment, suggesting that early intervention may help reverse the adverse effects of asthma-related inactivity on bone metabolism.

### 3.4 Differential skeletal risks of OCS and ICS

GCs are widely recognised as a leading cause of secondary osteoporosis, and their systemic use in asthma therapy represents a major threat to skeletal health (63). GCs markedly reduce bone matrix synthesis by directly promoting osteoblast and osteocyte apoptosis while simultaneously suppressing key osteogenic pathways, particularly Wnt/β-catenin signalling (64). During the early phase of asthma treatment, GCs upregulate RANKL expression and inhibit OPG production, thereby transiently enhancing osteoclast differentiation and activity and accelerating bone resorption (65). Early theories suggested GCs indirectly affected bone metabolism by targeting calcium-regulating organs like the kidneys and intestine, potentially causing secondary hyperparathyroidism. However, Frenkel et al. contend these indirect effects are not the primary osteoporotic mechanism, asserting direct actions on bone cells are responsible (66). A large-scale systematic comparison confirmed that, although the fracture risk associated with asthma itself is relatively low (OR = 1.06), OCS use significantly increases fracture risk in patients and shows a clear dose-dependent relationship. Specifically, after adjustment for sociodemographic factors and concomitant medications, OCS exposure increases fracture risk by 17% (OR = 1.17) (67). Bleecker et al. also noted that OCS are widely used in asthma management, and the risk of acute and chronic complications increases with cumulative dose (68). Another study further showed that fracture risk begins to increase even at doses as low as less than 5 mg/day of oral prednisone and rises with increasing cumulative exposure (69). Notably, the effects of different OCS formulations vary: oral prednisone is associated with a dose-dependent increase in overall fracture risk, whereas low-dose budesonide and hydrocortisone do not appear to increase fracture risk (70). After discontinuation, fracture risk declines rapidly, with a significant reduction within the first year. Overall fracture risk returns to that of the general population after approximately one year, although recovery of hip fracture risk is slower and remains elevated for about two years after cessation (71). These findings suggest that clinical practice should prioritize the lowest effective dose of OCS for the shortest possible duration, and that patients requiring long-term or repeated OCS therapy should undergo regular bone mineral density assessment, with anti-osteoporotic treatment introduced when appropriate. In contrast, ICS use, regardless of pattern (current, recent, or past use), was not significantly associated with fracture risk. This finding further highlights the specific risk profile of OCS in bone health management among patients with asthma (72).

As the cornerstone of asthma treatment, the long-term skeletal safety of ICS has remained a major clinical concern. However, studies examining the association between ICS use and fracture risk have yielded inconsistent findings. Although the systemic bioavailability of ICS is substantially lower than that of OCS, approximately 10-20% of the inhaled dose can still enter the systemic circulation and produce detectable serum concentrations. This pharmacokinetic characteristic suggests that ICS may exert skeletal adverse effects similar to those of OCS, which are recognised as the most common cause of secondary osteoporosis. OCS-related bone loss is closely associated with treatment dose and duration and can occur even during the early phase of therapy (73). Furthermore, substantial differences exist among individual ICS molecules. For example, fluticasone propionate exhibits high pulmonary deposition and low oral bioavailability, whereas systemic exposure primarily results from absorption of the inhaled fraction. It also demonstrates high plasma protein binding and a relatively long half-life. In contrast, budesonide undergoes rapid systemic clearance because of extensive first-pass metabolism and has a short half-life of approximately 2–3 hours. Ciclesonide, as a prodrug, exhibits low systemic exposure after activation within the lungs (74). These pharmacokinetic differences directly influence the potential skeletal risks associated with different ICS agents at conventional therapeutic doses. Available evidence indicates that long-term (≥2.7 years) use of ICS is associated with a 38% increased risk of osteoporotic fracture (OR = 1.38) and a 56% increased risk of hip fracture (OR = 1.56) (75). In paediatric patients, ICS use within 90 days prior to fracture significantly increases fracture risk (OR = 2.98) (5). In children with asthma receiving medium- to long-term, moderate-to-high-dose budesonide therapy, lumbar spine bone mineral density was significantly lower than that of the reference population. This association persisted even after excluding confounding factors such as short stature, suggesting that budesonide treatment may be associated with reduced bone mineral density (76). Systematic review evidence further suggests a potential overestimation of ICS effects on skeletal health. Neither fracture risk nor bone mineral density showed statistically significant differences between patients continuously using ICS for ≥12 months and controls. Across different study designs and age groups, the overall evidence indicates a favourable skeletal safety profile for ICS at conventional therapeutic doses (77). Specifically, among different ICS molecules, budesonide, due to its higher first-pass metabolism and short half-life, may exhibit better skeletal safety than fluticasone propionate at equivalent doses; however, this hypothesis still requires further validation. Furthermore, Alyasin et al. reported that the proportion of children with asthma exhibiting lower-than-expected bone mass (9.46%) did not differ significantly from that of the general population, and the duration of ICS use had no significant effect on bone mineral density (78).

In summary, existing evidence consistently confirms that OCS confer significant dose-dependent skeletal risks in patients with asthma. In contrast, evidence regarding the bone safety of ICS remains heterogeneous, and these inconsistencies are closely related to ICS type, dose, duration of use, and patient age. In clinical practice, ICS with favourable pharmacokinetic profiles should be preferred, and the lowest effective dose should be prescribed. Bone mineral density monitoring is recommended for patients requiring long-term moderate-to-high-dose therapy or for those with pre-existing osteopenia.

## 4 Conclusion and perspectives

This review systematically examines the increasingly recognized complex relationship between asthma and skeletal health. The findings indicate that this association extends beyond simple medication-related adverse effects and instead reflects a dual-hit model involving both intrinsic disease pathophysiology and external therapeutic factors. As a systemic inflammatory disease, asthma independently disrupts bone metabolism through mechanisms including Th17/Treg imbalance, proinflammatory cytokine release, and oxidative stress, thereby creating distinct skeletal risks. Regarding core therapeutic agents, GCs demonstrate differential skeletal effects. OCS confer significant dose-dependent risks of osteoporosis, whereas ICS appear to carry substantially lower skeletal risk despite heterogeneous evidence across studies. Nevertheless, optimization of inhaled therapy remains critically important for minimizing systemic corticosteroid exposure. In addition, two important intermediary factors, namely widespread VitD deficiency and asthma-related physical inactivity, further impair bone formation and repair. Through endocrine dysregulation and reduced mechanical loading, respectively, these factors jointly aggravate bone metabolic disturbances. Addressing this interdisciplinary challenge requires sustained efforts at multiple levels. Basic research should further elucidate the specific molecular mediators underlying lung-bone axis communication, with particular attention to the long-term effects of childhood asthma on skeletal development. In clinical practice, comprehensive strategies integrating inflammatory biomarkers and cumulative GCs exposure into risk prediction models, together with standardized bone health monitoring for high-risk populations, are needed to improve long-term skeletal outcomes in patients with asthma.

Based on the aforementioned effects of GCs on bone, comprehensive bone health monitoring strategies should be incorporated into the clinical management of patients with asthma. It is recommended that all patients receiving long-term GCs therapy, particularly OCS or moderate-to-high-dose ICS, undergo regular assessment of serum 25-Hydroxyvitamin D levels, with supplementation provided for those with deficiency (79). Regarding bone mineral density evaluation, Dual-energy X-ray Absorptiometry (DXA) remains the reference standard for osteoporosis diagnosis in the absence of definite osteoporotic fractures. Periodic DXA assessment should therefore be considered for patients receiving long-term OCS therapy, those exposed to repeated or continuous moderate-to-high-dose ICS for more than one year in combination with additional fracture risk factors such as advanced age, postmenopausal status, low body weight, or prior fracture history, as well as individuals with pre-existing osteopenia or osteoporosis (80). Furthermore, bone turnover markers including C-terminal telopeptide of type I collagen (CTX) and Procollagen type I N-terminal propeptide (P1NP) may provide dynamic assessment of bone metabolic activity in clinical research settings and can also serve as adjunctive monitoring tools in high-risk populations during routine practice (81). Collectively, these measures may facilitate earlier intervention for skeletal complications in patients with asthma and thereby reduce fracture risk.

Increasing attention has been directed toward natural products with anti-inflammatory and immunomodulatory properties, as mechanistic investigations of their active components may provide promising strategies for reducing dependence on both OCS and ICS. In recent years, natural product formulations have attracted growing interest because of their multi-component, multi-target, and synergistic effects. Certain natural products (such as *Modified-Xiaoqinglong* Decoction, *Wuwei Shaji* powder, Tetramethylpyrazine, ephedrine, etc.) have shown anti-inflammatory or immunomodulatory effects in asthma animal models and clinical observations, involving the Th17/Th17/Treg balance, the nuclear factor erythroid 2-related factor 2 (Nrf2) antioxidant pathway, and exosome-mediated mechanisms (82–85). However, current human evidence remains limited, and the above findings require validation through high-quality clinical studies. Increasing attention has been directed toward natural products with anti-inflammatory and immunomodulatory properties, as mechanistic investigations of their active components may provide promising strategies for reducing dependence on both OCS and ICS. In recent years, natural product formulations have attracted growing research interest because of their synergistic multi-component and multi-target effects.

In summary, a multidisciplinary approach integrating respiratory, paediatric and orthopaedic care with natural medicinal products can effectively reduce GCs dependence. Such integrated protocols support both asthma control and skeletal health management, thereby promoting holistic patient care.
